# Do large‐seeded herbs have a small range size? The seed mass–distribution range trade‐off hypothesis

**DOI:** 10.1002/ece3.3568

**Published:** 2017-11-19

**Authors:** Judit Sonkoly, Balázs Deák, Orsolya Valkó, Attila Molnár V., Béla Tóthmérész, Péter Török

**Affiliations:** ^1^ MTA‐DE Lendület Functional and Restoration Ecology Research Group Debrecen Hungary; ^2^ Department of Ecology University of Debrecen Debrecen Hungary; ^3^ MTA‐DE Biodiversity and Ecosystem Services Research Group Debrecen Hungary; ^4^ Department of Botany University of Debrecen Debrecen Hungary

**Keywords:** distribution area, light intensity, seed size, seed weight, soil fertility, soil moisture

## Abstract

We aimed to introduce and test the “seed mass–distribution range trade‐off” hypothesis, that is, that range size is negatively related to seed mass due to the generally better dispersal ability of smaller seeds. Studying the effects of environmental factors on the seed mass and range size of species, we also aimed to identify habitats where species may be at risk and need extra conservation effort to avoid local extinctions. We collected data for seed mass, global range size, and indicators for environmental factors of the habitat for 1,600 species of the Pannonian Ecoregion (Central Europe) from the literature. We tested the relationship between species’ seed mass, range size, and indicator values for soil moisture, light intensity, and nutrient supply. We found that seed mass is negatively correlated with range size; thus, a seed mass–distribution range trade‐off was validated based on the studied large species pool. We found increasing seed mass with decreasing light intensity and increasing nutrient availability, but decreasing seed mass with increasing soil moisture. Range size increased with increasing soil moisture and nutrient supply, but decreased with increasing light intensity. Our results supported the hypothesis that there is a trade‐off between seed mass and distribution range. We found that species of habitats characterized by low soil moisture and nutrient values but high light intensity values have small range size. This emphasizes that species of dry, infertile habitats, such as dry grasslands, could be more vulnerable to habitat fragmentation or degradation than species of wet and fertile habitats. The remarkably high number of species and the use of global distribution range in our study support our understanding of global biogeographic processes and patterns that are essential in defining conservation priorities.

## 1 INTRODUCTION

Studies of the question of rarity versus commonness usually aim to provide information that may help the conservation of rare species (Kunin & Schmida, 1997; Lavergne, Thompson, Garnier, & Debussche, 2004; Murray, Thrall, Gill, & Nicotra, 2002), and identifying plant traits that generally differ between rare and common species has long been an important aspect of ecological research (see Dostál, Fischer, & Prati, 2016; Fiedler, 1987; Murray et al., 2002). One thing which usually raises difficulties regarding the comparison of rare and common species is the fact that the term “rare” can be used to define different patterns, mainly describing narrowly distributed and/or sparsely populated species (Kunin & Gaston, 1993). However, there seem to be correlations between the different measures of rarity: Range size is usually positively related to local abundance (Brown, Stevens, & Kaufman, 1996; Köckemann, Buschmann, & Leuschner, 2009; Murphy, VanDerWal, & Lovett‐Doust, 2006) and to the diversity of habitats suitable for the species or niche breadth (Kolb, Barsch, & Diekmann, 2006; Slatyer, Hirst, & Sexton, 2013; Thompson, Gaston, & Band, 1999). One of the most frequently used measures of species rarity is geographical range size, which varies greatly among species (Lester, Ruttenberg, Gaines, & Kinlan, 2007). Range size is important in terms of conservation as it is related to extinction risk (Powney, Rapacciuolo, Preston, Purvis, & Roy, 2014; Walker & Preston, 2006), and the monitoring of range size is essential in the case of introduced and invasive species (Dostál et al., 2016; Gaston, 1994). In this study, we deal with range size and not with other aspects of rarity.

There are numerous possible general explanations to the great variance in species’ range sizes, such as (i) variance in environmental tolerance and/or habitat breadth (Kolb et al., 2006; Lloyd, Wilson, & Lee, 2003; Pither, 2003; Roukulainen & Vormisto, 2000; Slatyer et al., 2013), (ii) differences in dispersal ability (Edwards & Westoby, 1996; Lloyd et al., 2003; Van der Veken et al., 2007), (iii) evolutionary age (Guo, Brown, Valone, & Kachman, 2000; Webb & Gaston, 2000), and (iv) latitude of the geographical location (Morin & Chuine, 2006). In the case of plants (i) growth form or plant height (Kelly & Woodward, 1996; Murray et al., 2002; Roukulainen & Vormisto, 2000), (ii) seed size (Lavergne et al., 2004; Morin & Chuine, 2006; Procheş, Wilson, Richardson, & Rejmánek, 2012), (iii) seed production patterns (Peat & Fitter, 1994; Van der Veken et al., 2007), and (iv) seed longevity (Van der Veken et al., 2007) are also often considered to be related to range size. Despite the high number of potential explanations, a generally acceptable and supported hypothesis for this great variance has not been established yet (Lowry & Lester, 2006).

Range size is often hypothesized to be related to plants’ dispersal ability (Böhning‐Gaese, Caprano, van Ewijk, & Veith, 2006; Gaston & Kunin, 1997; Lloyd et al., 2003; Oakwood, Jurado, Leishman, & Westoby, 1993). Seeds with higher dispersal ability have a better chance to colonize new habitats than larger seeds with lower dispersal ability. On the other hand, species with poor dispersal capacity get adapted to local conditions more rapidly and thus speciate rapidly (Kunin & Gaston, 1997), which also results in smaller range sizes (Lester et al., 2007; Thompson et al., 1999). However, direct quantification of dispersal ability can be very difficult (Jacobson & Peres‐Neto, 2010); thus, different proxies are often used instead of a direct measure of it (Stewart et al., 1998; Tito de Morais et al., 2015). One of these proxies is seed size which is considered to be related to dispersal ability (Fenner & Thompson, 2005; Guo et al., 2000; Tremlová & Münzbergová, 2007), mostly due to the numerosity of small seeds (seed size/number trade‐off, Leishman, 2001) and the apparently obvious assumption that smaller seeds are more easily transported by wind and also by other agents (Greene & Johnson, 1993; Venable & Brown, 1988). Therefore, seed size can be used to estimate dispersal ability (Eriksson & Jakobsson, 1998; Herben, Nováková, Klimešová, & Hrouda, 2012; Westermann, von der Lippe, & Kowarik, 2011).

Despite the assumption that there is a negative relationship between seed size and range size, studies dealing with this question found contrasting results (Murray et al., 2002). The expected negative relationship between seed size and range size has already been demonstrated in previous studies (Guo et al., 2000; Morin & Chuine, 2006; Procheş et al., 2012; Walck, Baskin, & Baskin, 2001), but there are some counterexamples as well (Kolb et al., 2006; Lavergne, Garnier, & Debussche, 2003; Lavergne et al., 2004). One possible explanation for the lack of a general relationship is the fact that although smaller seeds have a greater chance to colonize new sites, they have a lower probability of survival there, which acts against range expansion (Fenner & Thompson, 2005). Because of these contrasting processes, a general relationship between seed size and range size has not been demonstrated yet, and it seems that this relationship is highly context‐dependent, varying from region to region (Geng et al., 2012).

Environmental conditions can have an effect on several plant traits, as these reflect the relationship of the plant with its environment (Chapin, Autumn, & Pugnaire, 1993; Geng et al., 2012). Thus, environmental factors may also determine range size, seed size, and their relationship. For example, seed size was found to be positively correlated with the shadiness of the habitat (Hodkinson et al., 1998; Metcalfe & Grubb, 1995; Salisbury, 1942). Soil parameters of the habitat can also be related to seed size: It was found to be negatively related to soil moisture and positively related to soil pH (Tautenhahn et al., 2008), and some studies reported larger seed size in habitats with infertile soils (Lee & Fenner, 1989; Liu et al., 2012). Available information on the relationship of environmental conditions and range size is restricted. For example, range size was found to be larger for plant species of aquatic and wetland habitats compared to species of terrestrial habitats (Ricklefs, Guo, & Qian, 2008). Despite the fact that narrow‐ranging species often inhabit infertile, stressed habitats (Fridley, Vandermast, Kuppinger, Manthey, & Peet, 2007; Hodgson, 1986; Thuiller, Lavorel, Midgley, Lavergne, & Rebelo, 2004), a negative relationship between soil fertility and range size has been found by Geng et al. (2012).

As no general relationships between seed size, range size, and environmental factors have been identified, and there is considerable variation in the results between different regions and ecosystems (Murray et al., 2002), our aim was to study these relationships on the herbaceous species of the Pannonian Ecoregion (Central Europe). The studied region has a diverse flora, being a good representative of the Central European flora, but it also has influences from other biogeographic regions (submediterranean, subatlantic, and continental influences, Fekete, Király, & Molnár, 2016). Thus, the studied region offers a unique opportunity to study the relationships between range size and seed size in a high number of species at a scale where these relationships are mostly detectable. We hypothesized that (i) seed mass is negatively related to range size, (ii) seed mass is related to environmental factors (soil moisture, light availability, and nutrient supply indicator values), and (iii) range size is related to environmental factors (soil moisture, light availability. and nutrient supply indicator values). Our ultimate goal was to reveal underlying mechanisms that shape the rarity of plant species.

## 2 METHODS

### 2.1 Data collection

At first, we obtained available thousand‐seed mass data (hereafter abbreviated as TSM; usually mentioned in the literature as TSW, i.e., thousand‐seed weight) using the checklist of plant species of the Pannonian Ecoregion (Central Europe). TSM values were mostly measured on seeds collected in the Pannonian Ecoregion (data obtained from the database of Török et al., 2013, 2016), data for some species of the Pannonian flora were obtained from databases or the literature (Csontos, Tamás, & Balogh, 2003, 2007; Schermann, 1967; LEDA Traitbase—Kleyer et al., 2008; SID—Liu, Eastwood, Flynn, Turner, & Stuppy, 2008). Note that in these databases—and also in the Török et al. (2013, 2016) database from where most of the records used in our analyses originate—usually the mass of the diaspore is given, but we use the term “seed mass” instead of “diaspore mass” for the sake of simplicity.

The quantification of global range for these species was based on the Flora database (Horváth et al., 1995), in which species of the Pannonian flora are categorized based on their distribution (see Table 1). The size of these distribution categories is expressed on a six‐grade ordinal scale, where increasing numbers indicate increasing range size. We categorized species based on these numbers, but in our analyses, we merged range size categories 1 and 2, as only one species (*Linum dolomiticum*) was classified into category 1. Thus, we had five distinct range size categories indicating increasing range (Table 1). For species for which distribution category could not be obtained from the Flora database because of missing data (34 species), we searched for range size data using other sources (eMonocot—http://www.emonocot.org; Encyclopedia of Life—http://eol.org; Global Biodiversity Information Facility—http://www.gbif.org; PESI Portal—http://www.eu-nomen.eu; Euro+Med PlantBase—http://www.emplantbase.org/home.html), and we classified species into the same distribution categories the Flora database uses based on these other sources. A similar approach of quantifying range size was used by Spitzer and Lepš (1988) and by Thomas (1991) and Spitzer, Novotny, Tonner, and Lepš (1993) for butterflies.

**Table 1:** Distribution types and range size categories based on distribution type, and the number of species in each type

Distribution	Range size category	Species number
Carpathian	1	10
Dacic	1	6
Illyric	1	5
Pannonic	1	37
Alpine–Balcanic	2	7
Balcanic	2	12
Central European	2	118
Central European ‐ Alpine	2	15
East Submediterranean	2	13
Pannonic–Balcanic	2	22
Pontic	2	40
Pontic–Mediterranean	2	79
Pontic–Pannonic	2	59
Turanian	2	8
Alpine	3	8
Atlantic ‐ Submediterranean	3	63
Boreal	3	7
Continental	3	89
European	3	167
Mediterranean	3	12
Sarmatian	3	5
Subatlantic	3	19
Submediterranean	3	138
Eurasian	4	400
Circumpolar	5	139
Cosmopolitan	5	122
Total	1–5	1,600

In order to improve the predictive value of the dataset for range effects, we omitted the following species groups from the analyses: (i) Woody species (except for chamaephytes and nanophanaerophytes, altogether 204 species) were excluded from the analyses, as seed size is strongly influenced by plant size (Díaz et al., 2016; Roukulainen & Vormisto, 2000; Thompson & Rabinowitz, 1989) and life form (Moles et al., 2005; Rockwood, 1985). (ii) All adventive species (altogether 337 species) were also excluded from the analyses as their distribution is strongly affected by human activities; thus, their recent range is not the result of their natural dispersal ability, and factors that determine indigenous and naturalized range can be considerably different (Procheş et al., 2012). (iii) We also excluded all aquatic plants (altogether 182 species) as the dispersal capacity of seeds dispersed by water is more likely to be determined by the buoyancy and the density of the seeds than by seed mass (Soons et al., 2016a), and they are often treated as a separate category in plant dispersal studies (Soons, Brochet, Kleyheeg, & Green, 2016b). Based on the analysis of Powney et al. (2014), we defined aquatic plants as species with a soil moisture indicator value >9 (Borhidi, 1995; Horváth et al., 1995). With the above‐mentioned restrictions, altogether 1,600 species were included in the analyses. Then, we obtained soil moisture, light intensity, and nutrient supply indicator values for these 1,600 species [based on Ellenberg indicator values F, L, and N (Ellenberg et al., 1992) modified and adapted for the Pannonian Ecoregion by Borhidi (1995); WB, LB, and NB, respectively]. Nomenclature follows Király (2009).

### 2.2 Statistical analyses

A generalized linear mixed model with Gaussian distribution and identity link was calculated for exploring the effect of species range, soil moisture, light intensity, and nutrient supply on the thousand‐seed mass of the studied species (GLMM; McCulloch & Searle, 2001). For the calculations, we log‐transformed the scores of thousand‐seed masses to improve normality of the dataset. As there was no phylogenetic tree available containing sufficient proportion of the species of the Pannonian flora with a small range size, performing a full phylogenetically informed analysis was not possible. To control for phylogenetic relatedness, we included “genus nested in family” as a random factor in the analyses (see Hanspach, Kühn, Pyšek, Boos, & Klotz, 2008; Koleček et al., 2014). We also fitted GLMMs with multinomial distribution and logit link function for studying the effect of soil moisture, light intensity, and nutrient supply on range size. In these analyses, we also used “genus nested in family” as a random factor. Then, we used Spearman's rank correlation to explore the direction and steepness of the relationships between each studied variable. All statistical analyses were performed using SPSS 20.0 program package.

## 3 RESULTS

Range size (*F *=* *4.613; *p *=* *.001), soil moisture (*F *=* *2.884; *p *=* *.001), light intensity (*F *=* *2.789; *p *=* *.007), and nutrient supply (*F *=* *2.978; *p *=* *.003) all had a significant effect on seed mass (TSM). Soil moisture (*F *=* *19.845; *p *<* *.001), light intensity (*F *=* *6.747; *p *<* *.001), and nutrient supply (*F *=* *14.273; *p *<* *.001) all had a significant effect on range size.

**Figure 1:**
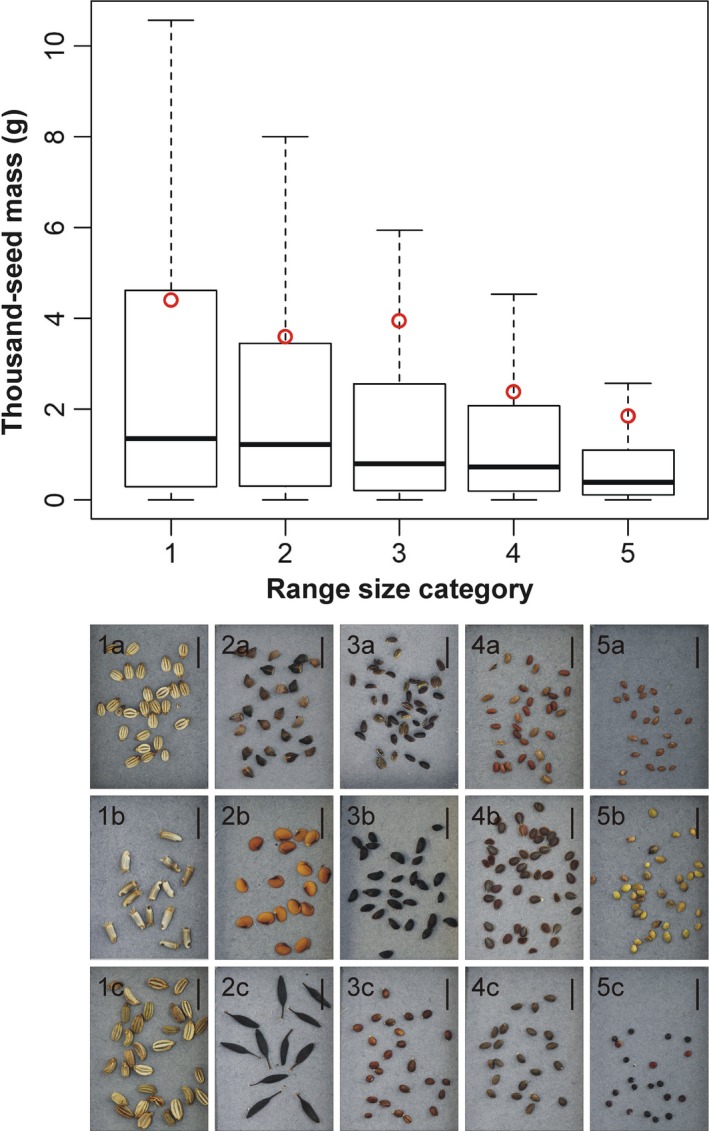
Boxplot of the thousand‐seed mass (TSM) of 1,600 species of the Pannonian flora belonging to different range size categories. Red circles represent the mean value. Under every box, the range size category is represented with seeds of three species from that category with TSM values close the median TSM of the given category. 1a—*Seseli osseum* (TSM = 1.2700 g); 1b—*Centaurea indurata* (TSM = 1.6217 g); 1c—*Seseli leucospermum* (TSM = 1.4630 g); 2a—*Echium maculatum* (TSM = 1.2270 g); 2b—*Biscutella laevigata* (TSM = 1.2250 g); 2c—*Lactuca quercina* (TSM = 1.2377 g); 3a—*Bupleurum praealtum* (TSM = 0.7673 g); 3b—*Vaccinium oxycoccos* (TSM = 0.8200 g); 3c—*Prunella grandiflora* (TSM = 0.7745 g); 4a—*Geranium dissectum* (TSM = 0.7287 g); 4b—*Lepidium perfoliatum* (TSM = 0.7187 g); 4c—*Marrubium peregrinum* (TSM = 0.7420 g); 5a—*Briza media* (TSM = 0.3893 g); 5b—*Ranunculus flammula* (TSM = 0.39130 g); 5c—*Chenopodium botrys* (TSM = 0.3920 g). All scale bars represent 5 mm

Rank correlations revealed that there is a significant but weak negative relationship between seed mass and range size (see Figure 1). We detected the strongest relationships between range size and soil moisture, range size, and nutrient supply: These environmental factors were significantly positively related to range size (Table 2). All other correlations were significant, but weaker (Table 2).

**Table 2:** Relationships between seed mass (thousand‐seed mass), range size and indicator values for soil moisture, light intensity, and nutrient supply (Spearman's rank correlation test)

	ρ	*p*
Seed mass		
Range size	−0.182	<.001
Soil moisture	−0.097	.001
Light intensity	−0.130	<.001
Nutrient supply	0.112	<.001
Range size		
Soil moisture	0.332	<.001
Light intensity	−0.095	<.001
Nutrient supply	0.290	<.001

## 4 DISCUSSION

Based on the analysis of the herbaceous species of the Pannonian Ecoregion, our results validated that there is a trade‐off between seed mass and range size in this Ecoregion, which may also exist globally. Although dispersal ability is probably a key factor in shaping plant distribution (Thompson & Ceriani, 2003), several other processes may act together in shaping the relationship between seed mass and range size. Smaller seeds have a better chance to reach distant sites due to their low mass and high number (Leishman, 2001; Venable & Brown, 1988). Small seeds also have a lower probability of being eaten by seed predators compared to large seeds, because smaller seeds may be less likely to be predated by vertebrate seed predators (Guo, Thompson, Valone, & Brown, 1995) and because they are less conspicuous than larger seeds accumulated in rather high density around the mother plant (Fenner & Thompson, 2005). Moreover, smaller seeds tend to persist longer in the seed bank (Thompson, Band, & Hodgson, 1993); therefore, they have a better chance to survive by bridging the temporarily unsuitable conditions of a potential new habitat and also to escape local extinction (Van der Veken, Bellemare, Verheyen, & Hermy, 2007). The link between narrow species range and large seed mass can be further enhanced by the fact that narrow‐ranging species usually have narrow habitat requirements (Lambdon, 2008; Slatyer et al., 2013), and for such species, larger seeds are more advantageous, as they are less likely to disperse away from the suitable habitat of the mother plant (Guo et al., 2000; but see Jacquemyn et al., 2007 and Jersáková & Malinová, 2007).

The trade‐off between seed mass and range size is well in accordance with some former results (Morin & Chuine, 2006; Procheş et al., 2012), while contradicts some others (Edwards & Westoby, 1996; Lavergne et al., 2004). The conflict between these results may arise from the fact that each study was conducted on different sets of species from different floras, regions, and ecosystems (Murray et al., 2002). However, to our knowledge, our study on the seed mass–range size relationship employs the highest number of species to date. To our knowledge, until now, the highest number of species employed to study this relationship was 234 (Morin & Chuine, 2006). The lack of detection of a relationship between seed size and range size reported by some studies can be explained by other factors that counteract the effect of better dispersal ability. For example, as the competition–colonization trade‐off predicts, seeds with better colonization ability have poorer competitive ability and a lower probability of successful establishment (Tilman, 1994). Moreover, one of the potential drawbacks of effective dispersal is that it implies that the seeds can get far away from the mother plant; hence, they have a bigger probability of reaching sites that are unsuitable, as the habitat of the mother plant is suitable by definition (Peat & Fitter, 1994). The evolutionary age of a species may also play an important role in determining its range size. Recently evolved species may have a narrow range even despite having good dispersal ability, simply because they have not had the opportunity to expand their range yet (Guo et al., 2000; Webb & Gaston, 2000).

Soil moisture was weakly negatively correlated with seed mass and positively with range size. This means that species characteristic to moist habitats have generally smaller seeds and bigger range size. These results are well in accordance with Baker (1972), who observed increasing seed size with increasing aridity of the habitat. However, according to Westoby, Jurado, and Leishman (1992), the very small seed size of wetland species is mostly responsible for this relationship. Our result corroborates this assumption, as we found smaller seeds for species that have high soil moisture values. Despite that we excluded all aquatic plants from our analyses, we could still observe a positive relationship between soil moisture and range size. This may be due to the fact that terrestrial plants with high soil moisture values are often characteristic to habitats nearly located to water bodies; thus, their seeds are often dispersed by water (Ozinga, Bekker, Schaminée, & van Groenendael, 2004; Soons et al., 2016a).

Light availability was negatively correlated to seed mass and only very weakly negatively correlated to range size. This means that species of shaded habitats have bigger seeds than that of open habitats, which has been demonstrated several times previously (Csontos, 1998; Hodkinson et al., 1998; Metcalfe & Grubb, 1995; Salisbury, 1942), and seems to be a rather general trend regardless to the studied biogeographic region. The finding that species of shaded habitats have bigger range size somewhat contradicts the result that species with bigger range size have smaller seeds, as smaller‐seeded species are associated with open habitats. However, the very weak negative correlation between light availability and range size could be due to wooded habitats in Europe have been more widely distributed than grasslands both historically and recently (Carboni, Dengler, Mantilla‐Contreras, Venn, & Török, 2015; Dengler, Janisová, Török, & Wellstein, 2014; Fischer & Wipf, 2002; Hobohm & Bruchmann, 2009).

Nutrient availability was positively correlated to both seed mass and range size, meaning that species of nutrient‐rich habitats have bigger seeds and are more widely distributed. On nutrient‐poor soils, seedlings of larger‐seeded species usually perform better than those of smaller‐seeded ones (Milberg & Lamont, 1997; Milberg, Péret‐Fernández, & Lamont, 1998). Some studies indeed found a negative relationship between seed size and nutrient supply (Lee & Fenner, 1989; Liu et al., 2012), but our results seemed to show an opposite trend for the Pannonian flora. This finding is in accordance with that of Grubb and Coomes (2008), who explained this with the supposition that plants on poorer soils have fewer resources to invest in the seeds.

Our results that species with a small range size have high light intensity and low soil moisture values corroborate the assumption that species of dry grasslands have high conservation value (Römermann, Tackenberg, Jackel, & Poschlod, 2008) and it is also supported by the finding that nearly twice as many endemic species of Europe are grassland specialist than forest specialists, even though forests are spatially much more extended than grasslands (Habel et al., 2013). Our finding that range size increases with increasing nutrient supply values is also in accordance with the general concept that common species are mostly associated with fertile, degraded habitats, while rare species are associated with less fertile and less disturbed ones (Hodgson, 1986). Ozinga et al. (2009) also stated that species of nutrient‐poor habitats are overrepresented among declining species. Powney et al. (2014) studied range change in the flora of Britain and found that species that prefer dry, infertile habitats are mostly associated with range decline, and similar patterns were found in Germany (Römermann et al., 2008). Ellenberg (1983) also demonstrated that species with high light and low nitrogen supply values, that is, those that are characteristic to open, infertile habitats such as dry grasslands, have the highest proportion of endangered ones (Diekmann, 2003).

Understanding the connection between species’ range size and other characteristics is getting more and more important in light of recent species extinctions and habitat fragmentation and degradation. Although this study is based on a single regional flora, the remarkably high number of species and the use of global range make our results relevant for several ecosystems and support our understanding of global biogeographic processes and patterns that are essential in defining conservation priorities.

## DATA ACCESSIBILITY

All data used in the analyses are available from Dryad—DOI: https://doi.org/10.5061/dryad.244sn.

## CONFLICT OF INTEREST

None declared.

## AUTHORS’ CONTRIBUTIONS

AMV, PT, BT, and JS conceived and designed the study; OV and JS collected the data; BD, JS, and PT performed the analyses; JS led the writing with contributions from all authors.
